# Optimized method for segmentation of ancient mural images based on superpixel algorithm

**DOI:** 10.3389/fnins.2022.1031524

**Published:** 2022-11-02

**Authors:** Jinxing Liang, Anping Liu, Jing Zhou, Lei Xin, Zhuan Zuo, Zhen Liu, Hang Luo, Jia Chen, Xinrong Hu

**Affiliations:** ^1^School of Computer Science and Artificial Intelligence, Wuhan Textile University, Wuhan, Hubei, China; ^2^Engineering Research Center of Hubei Province for Clothing Information, Wuhan, Hubei, China; ^3^Hubei Province Engineering Technical Center for Digitization and Virtual Reproduction of Color Information of Cultural Relics, Wuhan, Hubei, China; ^4^School of Communication, Qufu Normal University, Rizhao, Shandong, China

**Keywords:** ancient murals, image segmentation, SLIC, superpixel, density-based clustering, k-means clustering

## Abstract

High-precision segmentation of ancient mural images is the foundation of their digital virtual restoration. However, the complexity of the color appearance of ancient murals makes it difficult to achieve high-precision segmentation when using traditional algorithms directly. To address the current challenges in ancient mural image segmentation, an optimized method based on a superpixel algorithm is proposed in this study. First, the simple linear iterative clustering (SLIC) algorithm is applied to the input mural images to obtain superpixels. Then, the density-based spatial clustering of applications with noise (DBSCAN) algorithm is used to cluster the superpixels to obtain the initial clustered images. Subsequently, a series of optimized strategies, including (1) merging the small noise superpixels, (2) segmenting and merging the large noise superpixels, (3) merging initial clusters based on color similarity and positional adjacency to obtain the merged regions, and (4) segmenting and merging the color-mixing noisy superpixels in each of the merged regions, are applied to the initial cluster images sequentially. Finally, the optimized segmentation results are obtained. The proposed method is tested and compared with existing methods based on simulated and real mural images. The results show that the proposed method is effective and outperforms the existing methods.

## Introduction

Ancient murals have been eroded over time by different natural factors such as light, temperature, humidity, carbon dioxide, and bacteria (Shi and Lu, 2005), resulting in the degradation of their appearance, particularly their color. The historical value of these ancient murals may get hidden by the fading colors. Current restoration works can help rediscover the hidden information. In the aspect of restoration for ancient murals, the traditional imitation method is easily influenced by personal experience, and the imitation work is irreversible. Fortunately, computer-aided digital restoration can help overcome the shortcomings of traditional imitation restoration. Furthermore, digitally archived files can be more easily displayed, reproduced, and permanently stored.

The high-precision segmentation of ancient mural images is the foundation of digital virtual restoration. With the support of image processing technology and the collected database of ancient murals (Liang et al., 2016; Jin-xing and Xiao-xia, 2017), the faded color can be restored with high fidelity. However, ancient mural images are very complex in terms of color because of the different color degradation conditions existing in these murals, such as fading, wearing, and shedding (Lu et al., 1998), which make it difficult to achieve high-precision segmentation using traditional segmentation algorithms. For example, current segmentation methods cannot achieve color consistency and positional connectivity when segmenting an ancient mural image.

In the past decades, a few human–computer interaction-based image segmentation methods have been proposed based on traditional image segmentation algorithms. Some intelligent human–computer interactive image segmentation systems have been developed (Hua et al., 2002), including region growth-based (Baogang et al., 1999) and edge detection-based (Pan and Lu, 2003). Lu et al. (2002) attempted to automatically extract the region to be repaired using a color histogram; however, the method is only applicable to the extraction of local regions with consistent colors. Li et al. (2000) proposed an image segmentation method that combined edge detection and region growth technologies.

With the rapid development of deep learning technology, image segmentation based on semantic or instance information of images has made great progress. Among the current deep learning-based image segmentation methods, the representative one is the full convolutional network (FCN) proposed by Long et al. (2015), which uses up-sampling instead of a full connection layer and can process images of different sizes. Ronneberger et al. (2015) proposed the U-net framework, which is suitable for running smaller batch dataset samples. Badrinarayanan et al. (2017) proposed the SegNet neural network, which greatly reduced the model parameters and improved the efficiency. Zhao et al. (2017) proposed PspNet, which uses global feature prior knowledge to analyze the different scenes and realize semantic segmentation. Lin et al. (2017) proposed a Re-fineNet framework using chain residual connection. Nevertheless, as it is exceptionally difficult to build a large and robust database to train the mentioned frameworks, they are unsuitable for the segmentation of ancient mural images. Therefore, most current methods are still based on traditional image segmentation algorithms, such as threshold-based (Otsu, 1978; Kapur et al., 1985; Yen et al., 1995), edge detection-based (Rosenfeld, 1981; Er-Sen et al., 2009), and clustering-based (Dasgupta, 2008; Yan et al., 2012).

Although traditional image segmentation algorithms are used extensively in different areas, owing to the complexity of the color appearance of mural images, they are not directly applicable to ancient mural images without human assistance. For example, simple or multiple thresholds cannot deal with the color-mixing problems in ancient murals for threshold-based methods (Otsu, 1978; Kapur et al., 1985; Yen et al., 1995). Further, the edge detection-based segmentation algorithm (Rosenfeld, 1981; Er-Sen et al., 2009) can only obtain an incomplete local contour of the input image. In addition, for clustering-based image segmentation algorithms, k-means or fuzzy c-means clustering (FCM) algorithms (Dasgupta, 2008; Yan et al., 2012) perform global clustering on the input image that only meets the coarse color consistency and cannot realize the positional connectivity of their clustering results. The limitations of the current methods for segmenting ancient mural images are apparent.

To address the current challenges in ancient mural image segmentation, an optimized method based on a superpixel algorithm is proposed in this study. First, the SLIC algorithm (Achanta et al., 2012) was applied to the input mural images to obtain the superpixels. The DBSCAN algorithm (Bäcklund et al., 2011; Kovesi, 2013) was then used to cluster the superpixels to obtain the initial clustered images. However, the noisy superpixels in the initial clustering result hinder the color consistency and positional connectivity of the initial clustering of the superpixel image. Therefore, a series of optimized strategies were implemented to deal with the noisy superpixels, including (1) merging the small noise superpixels, (2) segmenting and merging the large noise superpixels, (3) merging the initial clusters based on color similarity and positional adjacency to obtain the merged regions, and (4) segmenting and merging the color-mixing noisy superpixels in each of the merged regions. Finally, the optimized segmentation results were obtained. The experiments showed that the proposed method was effective and outperformed the existing methods in segmenting ancient mural images.

## Materials and methods

### Simple linear iterative clustering algorithm

The SLIC algorithm is used extensively for image processing (Achanta et al., 2012). For the SLIC algorithm, the input image is first converted to the CIELAB color space, and for each pixel in the image, a five-dimensional feature vector (*l*, *a*, *b*, *x*, *y*) is constructed by combining the color features (*l*, *a*, *b*) and the spatial features (*x*, *y*). During the clustering process, the number of superpixels *K* must be set, and the initial cluster centers *C*_*k*_ = [*l*_*k*_, *a*_*k*_, *b*_*k*_, *x*_*k*_, *y*_*k*_] (*k* = 1, 2, …, *K*) can be acquired according to the step value *S* of the regular grid. Based on the feature similarity between the initial cluster centers and all the pixels, each image pixel is assigned to the most similar cluster center within the area of *2S* × *2S*. After the first round of clustering, the cluster centers are updated based on the first clustering result, and the update is repeated until the cluster centers are stable or the maximum update number is reached. The feature similarity between the cluster centers and each pixel consists of two parts, where the color and spatial similarities are calculated using Equations 1 and 2, respectively.


(1) $$\begin{array}{c} d\,i\,s\,t_{c\,o\,l\,o\,r}=\sqrt{{\left(l_{k}-l_{i}\right)}^{2}+{\left(a_{k}-a_{i}\right)}^{2}+{\left(b_{k}-b_{i}\right)}^{2}} \end{array}$$

(2) $$\begin{array}{c} d\,i\,s\,t_{s\,p\,a\,c\,e}=\sqrt{{\left(x_{k}-x_{i}\right)}^{2}+{\left(y_{k}-y_{i}\right)}^{2}} \end{array}$$

where *l*_*i*_, *a_*i*_*, and *b*_*i*_ represent the *L*, *a*, and *b* values of the ith pixel in the CIELAB color space, respectively, *l*_*k*_, *a_*k*_*, and *b*_*k*_ represent the *L*, *a*, and *b* values of the kth cluster center, respectively, *x*_*k*_ and *y*_*k*_ represent the coordinates of the kth cluster center, and *x*_*i*_ and *y*_*i*_ represent the coordinates of the ith pixel. With the two different similarities between the cluster centers and each of the image pixels, their formal feature similarity is defined in Equation 3:


(3) $$\begin{array}{c} d\,i\,s\,t_{s\,i\,m\,i\,l\,a\,r\,i\,t\,y}=\sqrt{{\left(d\,i\,s\,t_{c\,o\,l\,o\,r}\right)}^{2}+{\left(\frac{d\,i\,s\,t_{s\,p\,a\,c\,e}}{S}\right)}^{2}\,\lambda^{2}} \end{array}$$

where *S* = sqrt((*m* × *n*)/*K*) represents the size of the initialized superpixels, in which *m* and *n* are the width and height of the input image, respectively, and *K* is the desired number of superpixels, and λ is the weight constant of spatial proximity, which directly influences the proportion of the spatial proximity in the formal feature similarity. Usually, the larger the λ value, the more regular the superpixels generated by the SLIC algorithm; however, the lower the fit with the image boundary. In contrast, the smaller the λ value, the more irregular the superpixels but a high degree of fit with the boundary of the image.

### Density-based spatial clustering of applications with noise algorithm

The DBSCAN algorithm (Bäcklund et al., 2011) is used to cluster the superpixels generated by the SLIC algorithm. It can connect density-reachable superpixels to a large cluster. The sample dataset is set as *D* = (*x*_1_, *x*_2_, …, *x*_*n*_). The DBSCAN algorithm divides the data points into core, boundary, and noise points according to a set of neighborhood values (ε, *Minpts*), where ε is the neighborhood radius and *Minpts* is the sample density threshold.

The principle of DBSCAN is as follows: First, for the input dataset *D* = (*x*_1_, *x*_2_, …, *x*_*n*_), the values of ε and *Minpts* are set as described previously, and the number of data points in *D* is initialized to obtain the label array. Then, the method selects a data point *x*_*i*_ (*i* = 1, 2, …, *n*) from D, where the data point *x*_*i*_ in *D* includes the features of the superpixels generated by the SLIC. Third, according to the values of ε and *Minpts*, the selected data point is judged as the core point. If the data point is a core point, then all the data points that are connected to the core points are identified and labeled together as a large cluster. If the selected data point is not a core point, it is judged as a boundary or noise point, and the corresponding process is applied to the data point. Finally, when all the data points in *D* are processed, the initial clustering result is obtained for the superpixels in the image. Additional details regarding the DBSCAN algorithm can be found in the literature (Bäcklund et al., 2011).

### Proposed method

**FIGURE 1:**
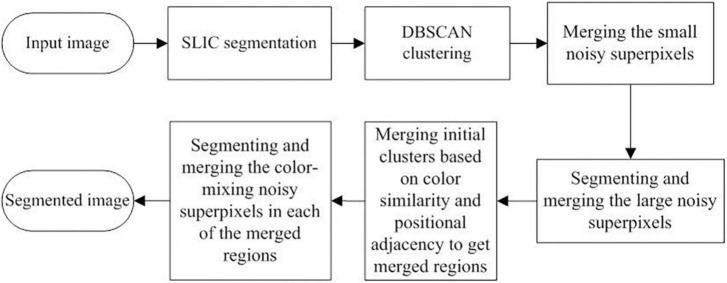
Workflow of the proposed optimized method for ancient mural image segmenting.

To address the limitations of the SLIC and DBSCAN algorithms in segmenting ancient mural images, an optimized method for the segmentation of ancient mural images based on the superpixel algorithm is proposed in this study. The proposed method aims to realize color and spatial consistency at the same time when segmenting mural images, where color consistency means that different subregions with the same color in a large region should have the same label and spatial consistency means that the degraded subregions should merge into the large region to which they belong. The workflow of the proposed optimized method is shown in Figure 1.

First, the input image was transformed to a superpixel image based on the SLIC algorithm, followed by the DBSCAN algorithm to obtain the initial clustering result. Then, for the different types of noise superpixels in the initial clustering result, a series of optimized strategies were sequentially applied to the initial cluster images. These optimized strategies include: (1) merging the small noise superpixels, (2) segmenting and merging the large noise superpixels, (3) merging the initial clusters based on color similarity and positional adjacency to obtain the merged regions, and (4) segmenting and merging the color-mixing noisy superpixels in each of the merged regions. Finally, the optimized segmentation results were obtained. The specific implementation steps are as follows:

Step 1: The input image was segmented using the SLIC algorithm to obtain the regular and compact superpixels. Simultaneously, the label matrix *L* of the superpixel image and the adjacent matrix *Am*, describing the adjacency relation among the superpixels, were obtained.

Step 2: The superpixel image is clustered using the DBSCAN algorithm to obtain the initial clustering results. Four types of clusters exist in the initial clustering results: the small noise superpixels that did not cluster with any other superpixels, the large noise superpixels that did not cluster with any other superpixels, the normal cluster with pure color, and the abnormal cluster with color-mixing superpixels on the edge.

Step 3: The small noise superpixels are extracted and merged into the adjacent cluster with the best color similarity.

Step 4: For large noise superpixels, if they are pure color, they are merged into the adjacent cluster that has the best similarity. If they have more than one color in them, they are first segmented using the k-means algorithm and then merged into the adjacent cluster with the best color similarity.

Step 5: After steps 3 and 4, the initial clusters are further merged according to their color similarity and spatial adjacency. Thus, relatively large subregions (called merged regions) are obtained consisting of the processed initial clusters.

Step 6: After Step 5, the edges of each merged region are traversed to determine whether there are mixed-color superpixels. If so, they are processed according to the method in Step 4, i.e., they are segmented and merged into the adjacent cluster with the best color similarity.

**FIGURE 2:**
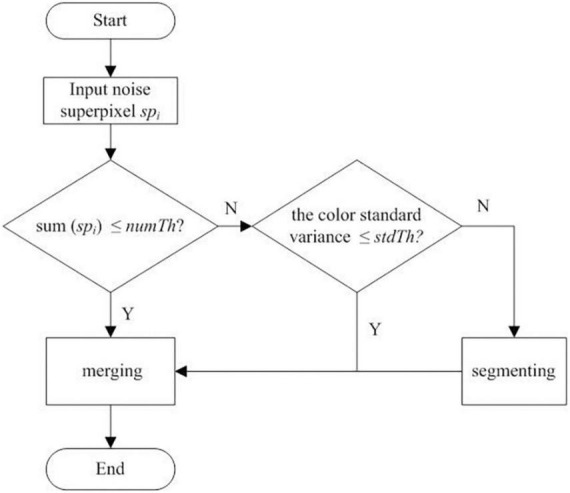
Workflow to deal with the small and large noise superpixels of the initial clustering result.

Finally, the optimized segmentation results are obtained. In the proposed optimized method, the definition and classification of noise superpixels are based on the threshold *number Th*. If the total number of pixels in a superpixel is less than *numTh*, it is classified as a small noise superpixel. If it is greater than the preset threshold *numTh*, it is classified as a large noise superpixel. The workflow to deal with small noise (Step 3) and large noise (Step 4) superpixels is plotted in Figure 2. The workflow details are as follows:

(1) Merging small noise superpixels. First, the empirical parameter *numTh* is set to process small noise superpixels. The condition to judge whether the target superpixels are small is to judge whether sum(*sp*_*i*_) ≤ *numTh*, where sum(*sp*_*i*_) represents the total number of pixels in the superpixel *sp*_*i*_. If it is, all the adjacent superpixel *neighbors* = {*nsp*_1_, *nsp*_2_, …, *nsp*_*t*_} to *sp*_*i*_ are selected based on the adjacent matrix *Am* (acquired in the SLIC stage), where *nsp*_1_, *nsp*_2_, …, *nsp*_*t*_ are the superpixels satisfying the adjacency conditions to *sp*_*i*_, the subscript represents the index of each adjacent superpixel in *the neighbors*, and *t* represents the number of superpixels adjacent to *sp*_*i*_.

Then, the color similarity is calculated between each of the adjacent superpixels in the neighbors and *sp*_*i*_ according to Equation 4, and the corresponding similarity *dist* = {*dist*_1_, *dist*_2_, …, *dist*_*t*_} is obtained, where the best superpixels matched to the small noise superpixels can be found using Equation 5. Thus, the small noise superpixels are merged into the corresponding cluster where the matched superpixels are.


(4) $$dist_{color}\left(sp_{i},sp_{j}\right)\;=\;\sqrt{\left(\bar{l_{\dot{l}}}-\bar{l_{\dot{j}}}\right)+\;\left(\bar{a_{\dot{l}}}-\bar{a_{\dot{j}}}\right)+\;(\bar{b_{\dot{l}}}-\bar{b_{\dot{j}}})}$$

(5) $$\begin{array}{c} i\,n\,d^{*}=\text{arg }{m\,i\,n}_{i\,n\,d}\left(d\,i\,s\,t\right) \end{array}$$

where *l*_*k*_, *a*_*k*_, *and b_*k*_* (*k* = *i*, *j*) are the mean color values of all the pixels in superpixel *sp*_*k*_, *ind* represents the index of the elements in *dist*, and ind* denotes the index value *ind* corresponding to the smallest *dist* value. Finally, the target superpixels are assigned to the cluster in which the matched superpixels are, and the merging of small noise superpixels is completed.

(2) Segmenting and merging the large noise superpixels. As shown in Figure 2, if the color standard deviation of the large noise superpixel *sp*_*i*_ is less than the set threshold *stdTh*, the similarity merging process is performed on *sp*_*i*_. First, according to Equation 4 and the adjacent matrix *Am*, the superpixel set *neighbors* adjacent to the large superpixel *sp*_*i*_ are obtained. Then, according to formula (6), the superpixel subset *subneighbors* satisfying similarity merging are obtained. The superpixels in the *subneighbors* are not only adjacent to the *sp*_*i*_ in the spatial position but also consistent with the *sp*_*i*_ in color. Finally, the array index ind* corresponding to the minimum value in *dist* is obtained according to Equation 5, and *sp*_*i*_ is divided into clusters to which *sp* belongs according to the obtained ind* to complete the similarity merging of the large noise superpixels.


(6) $$\begin{array}{c} \left\{ \begin{array}{c} subneighbors=\{x|x\in neighbors\cap dist_{c\,o\,l\,o\,r}\;(x,sp_{i})\\ <simTh\}\;dist=\{d|d=dist_{c\,o\,l\,o\,r}\;(x,sp_{i})\;,\;x\in subneighbors\} \end{array}\right. \end{array}$$

**FIGURE 3:**
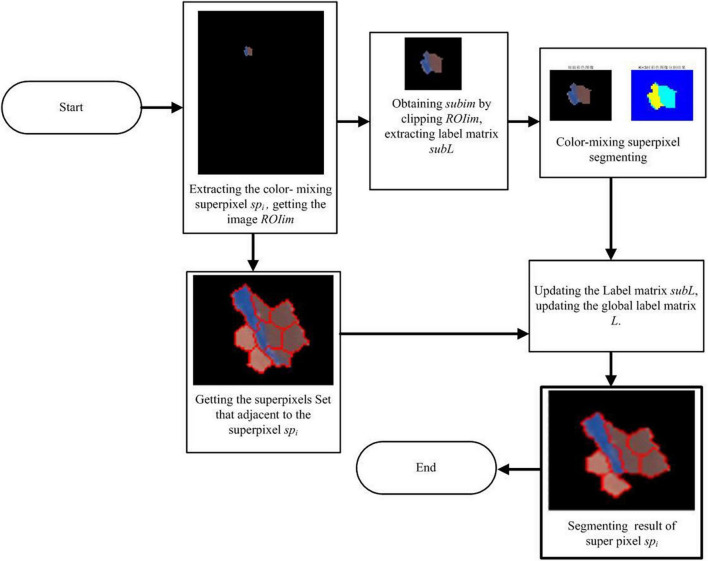
Workflow of color-mixing superpixels segmenting.

However, if the color standard deviation of the large noise superpixels is greater than the preset threshold *stdTh*, it indicates that it is a color-mixing noise superpixel, and the superpixels need to be segmented before merging into adjacent clusters. The workflow of the color-mixing superpixel segmentation is shown in Figure 3. The details of the segmentation of the color-mixing superpixels are as follows:

1. The region of the interest image and label matrix including the color-mixing superpixels *sp*_*i*_ are extracted. A rectangular frame centered on the color-mixing superpixel coordinates is set, and the image *ROIim* containing the target superpixels is cropped to obtain the cropped image *subim*. Simultaneously, a rectangular frame is used to extract the label matrix of *ROIim* to obtain the local label matrix *subL* that corresponds to the cropped image *subim*, which contains the labels of the superpixel *sp*_*i*_.
2. The k-means algorithm is used to segment the cropped image *subim*. The input image *subim* is divided into a black background region *k*_1_, color-mixing superpixel dichotomous region *k*_2_, and *k*_3_ by setting the segmentation parameter *K* to 3, where *k*_1_, *k*_2_, and *k*_3_ are the labels of each segmentation subregion, which belong to {1, 2, 3} and satisfy *k*_1_ ≠ *k*_2_ ≠ *k*_3_.
3. The label matrix *subL* is updated according to the image segmentation result of *the subim*. First, the dataset of neighbor superpixels of the large noise superpixel *sp*_*i*_ is established based on the superpixels adjacent to the matrix *Am*, and then, the subregions *k* (*k* = *k*_1_, *k*_2_, *k*_3_) of the *subim*age are extracted. If *k* is equal to *k*_1_, it indicates that the extracted subregion corresponds to a black background, and there is no need to update the label matrix *subL*. If the value of *k* is equal to *k*_2_ or *k*_3_, the most similar adjacent superpixels *sp* to subregion *k* will be determined based on Equations 5 and 6, and subregion *k* will be merged into *sp* by updating its label matrix, where the label matrix *subL* is updated automatically. After the label matrix *subL* is updated, the corresponding area in the label matrix *L* is replaced by the *subL*, and the segmenting and merging of the large noise superpixels is completed.

(3) Merging the initial clusters based on color similarity and positional adjacency to obtain merged regions. The DBSCAN algorithm for superpixel clustering divides *K* superpixels *D* = {*sp*_1_, *sp*_2_, …, *sp*_*K*_} into *n* clusters, namely, *D*_1_, *D*_2_, …, *D*_*n*_ (*n* < *K*), and satisfies the following conditions:


(7) $$\begin{array}{c} \left\{ \begin{array}{c} |D_{i}|=1\left(i=1,2,\;\cdots \;,n\right)\\ D_{i}\cap D_{j}=\emptyset \left(i\neq j\right)\cap \sum_{1}^{n}|D_{i}|=K \end{array}\right. \end{array}$$

where *D*_*i*_ represents the ith cluster and |*D*_*i*_| represents the number of superpixels in the cluster. The merging of clusters in this stage includes two conditions: (1) merging the clusters that initially contain a certain number of superpixels and (2) merging the adjacent clusters based on the chain propagation theory. The first condition aims to merge the small clusters to the adjacent clusters to obtain relatively large clusters, after which the clusters are merged into a large merged region based on the chain propagation theory. The method for the first condition is as follows:

1. Set the number of superpixels in the clusters to *numTh_s* as the threshold and use it to filter out all clusters that meet the conditions *C* = {*D*_*i*_ | 1 < | *D*_*i*_| ≤ *numTh_s*}.
2. Choose a cluster *D*_*i*_ in *C* and traverse each superpixel *spi* in cluster *D*_*i*_, using the principal of Equations 5 and 6 to determine the most similar superpixels *sp* that do not belong to *D*_*i*_.
3. If *sp* exists, determine the corresponding cluster *D*_*j*_ where superpixels *sp* are located, then merge the cluster *D*_*i*_ into cluster *D*_*j*_, and set *D*_*i*_ to null.
4. Steps 2–3 are repeated until all the clusters are processed. The implementation of the pseudocode for the first merge condition in MATLAB language is listed in Table 1.

**TABLE 1:** Implementation of pseudocode in MATLAB language on merging the clusters that contain a certain number of superpixels.

**Algorithm 1**: Merging of clusters containing a specific number of superpixels	
**Input:**	cluster set *C* = {*D*_1_, *D*_2_, …, *D*_*n*_}, adjacent matrix *Am*, threshold of number of superpixels *numTh*.
**Output:**	new cluster set *C*_1_ = {*D*_1_, *D*_2_, …, *D*_*m*_}.
1.	**for** *i* = 1:length(*C*)
2.	*D*_*i*_ = *C*{*i*};
3.	**if** 1 < | *D*_*i*_| < = = *numTh* then
4.	**for** *j* = 1:| *D*_*i*_|
5.	*sp*_*i*_ = *D*_*i*_(*j*);
6.	Find *neighbors* of *sp*_*i*_ according to *Am*;
7.	Compute *dist* between *sp*_*i*_ and neighbor in *neighbors*;
8.	Find out the superpixels *sp* most similar to *sp*_*i*_ according to the minimum value of *dist* and find *D*_*j*_ to which *sp* belongs;
9.	**if** *sp* ! = _∅_ **then**
10.	*D*_*j*_ = *D*_*i*⋃_ *D*_*j*_;
11.	*D*_*i*_ = _∅_;
12.	**end if**
13.	**end for**
14.	**end if**
15.	**end for**
16.	Remove empty set in the set *C* and reconstruct set *C* to get *C*_1_;

Following the first condition, the algorithm for merging the adjacent clusters based on the chain propagation theory is applied to the clusters. The algorithm regards each cluster as a node, randomly selects an unprocessed cluster, *D*_*i*_, and takes this cluster as the starting cluster. Then, the algorithm connects all the unprocessed clusters with the same density and color as cluster *D*_*i*_ on a certain path and integrates them into larger clusters, which is termed as the merged region. The key steps of the algorithm are as follows:

1. Input the cluster dataset *C*_1_ = {*D*_1_, *D*_2_, …, *D*_*m*_}, where *m* < *n*.
2. Remove one of the unprocessed clusters *D*_*i*_ (*i* = 1, 2, …, *m*).
3. Input all the superpixels in cluster *D*_*i*_ into array *S* and input the label mark of cluster *D*_*i*_ in *ind*.
4. Remove one superpixel *sp*_*i*_ from *S* and determine the superpixels that set the *neighbors* adjacent to *sp*_*i*_.
5. Use the similarity rules defined in Equations 5 and 6 to determine the super pixel *sp* that does not belong to *D*_*ind*_ but is most similar to *sp*_*i*_.
6. If *sp* does not exist and there are superpixels in array *S* that have not been traversed, proceed to step 4. If *sp* does not exist and the superpixels in array *S* have been traversed, proceed to step 2. If *sp* exists, then find the cluster *D*_*j*_ where *sp* is located, copy the superpixels in cluster *D*_*j*_ into array *S*, merge the superpixels in *D*_*ind*_ into cluster *D*_*j*_, then set *D*_*ind*_ empty and assign the index *j* of cluster *D*_*j*_ to *ind*, and return to step 4.
7. Repeat steps 2–6 until all clusters are processed, and finally, complete the merging of clusters to obtain the merged regions. The implementation of the pseudocode of the second merge condition in MATLAB language is listed in Table 2.

**TABLE 2:** Implementation of pseudocode in MATLAB language on merging the clusters based on chain propagation theory.

**Algorithm 2**: Merging of clusters based on chain propagation theory	
**Input:**	cluster set *C*_1_ = {*D*_1_, *D*_2_, …, *D*_*m*_}, adjacent matrix Am.
**Output:**	new cluster set *C_2_* = {*D*_1_, *D*_2_, …, *D*_*q*_}.
1.	**for** *i* = 1:length(*C*_1_)
2.	*ind* = *i*;
3.	*S* = *C*{*i*}; Mark *D*_*ind*_ as processed;
4.	**while** *i* > 0
5.	*flag* = 0;
6.	**for** *j* = 1:length(*S*)
7.	*sp*_*i*_ = *S*(*j*);
8.	Find *neighbors* of *sp*_*i*_ according to *Am*;
9.	Compute *dist* between *sp*_*i*_ and neighbor in *neighbors*;
10.	Find out the superpixels *sp* most similar to *sp*_*i*_ according to the minimum value of *dist* and find *D*_*j*_ to which *sp* belongs.
11.	**if** s*p* ! = _∅_ **then**
12.	*flag* = 1;
13.	*S* = *D*_*j*_;
14.	*D*_*j*_ = *D*_*ind*⋃_ *D*_*j*_;
15.	*D*_*ind*_ = _∅_;
16.	*ind* = *j*; Mark *D*_*ind*_ as processed;
17.	break;
18.	**end if**
19.	**end for**
20.	**if** *flag* = = 0 **then**
21.	break;
22.	**end if**
23.	**end while**
24.	**end for**
25.	Remove empty set in the set *C*_1_ and reconstruct set *C*_1_ to get *C*_2_;

(4) Segmenting and merging the color-mixing noisy superpixels in each merged region. As illustrated in Figure 1, four types of clusters exist in the initial clustering results, where an abnormal cluster with color-mixing superpixels on the edge is one type. However, these edge color-mixing superpixels still exist in the merged region as no step is required to process them in the above works. Therefore, it is necessary to address this issue for the high-precision segmentation output of ancient mural images. The method to process the edge color-mixing superpixels in the merged regions is the same as in Figure 3; the method is not repeated here.

## Experiment and results

### Experiment settings

To verify the effectiveness of the proposed optimized method, the hardware environment of the experiment was configured using a desktop computer with an Intel (R) core (TM) i7-9700 processor and a Windows 10 operating system, and the programming environment was MATLAB R2019a. The data for the test algorithm were a simulated Dunhuang mural image of 1,173 × 829 pixels and two real Dunhuang mural images of 554 × 694 and 806 × 1120 pixels. The methods in the literature (Dasgupta, 2008; Yan et al., 2012; Kovesi, 2013) were compared to verify the superiority of the proposed method.

### Evaluation metrics

The image segmentation quality evaluation metrics are divided into supervised and unsupervised methods. In view of the complex colors in the Dunhuang mural images, it is difficult to develop a reasonable database of fine-segmented mural images. Therefore, this study used unsupervised evaluation metrics (Hui et al., 2008) to quantitatively analyze the segmentation performance of the tested methods. The unsupervised segmentation evaluation metric *F* was based on the color variance to measure the performance of the segmentation algorithm. It calculates the color square error of each segmentation region and uses the square root of the total number of segmentation regions as a weight to punish over-segmentation. The *F* error is calculated as follows:


(8) $$\begin{array}{c} \left\{ \begin{array}{c} F=\sqrt{N}\,\sum_{j=1}^{N}\frac{e_{x}^{2}\,\left(R_{j}\right)}{\sqrt{S_{j}}}\\ e_{x}^{2}\,\left(R_{j}\right)=\sum_{p\in R_{j}}{\left(C_{x}\,\left(p\right)-\frac{\sum_{p\in R_{j}}C_{x}\,\left(p\right)}{S_{j}}\right)}^{2} \end{array}\right. \end{array}$$

where *C*_*x*_(*p*) represents the feature values of the pixel *p* in terms of color *x*, *S*_*j*_ represents the number of pixels in region *j*, *R*_*j*_ represents the set of pixels in region *j*, and *N* represents the number of regions of the entire segmented image. The smaller the *F* value, the better the performance of the segmentation algorithm, and vice versa.

Another evaluation metric *E* is entropy-based, which is an evaluation metric developed by combining the information theory and the minimum description length (MDL) principle. It defines the regional entropy as a measure of the consistency within the region and defines the layout entropy to punish the situations when the entropy within the region is small. Given a segmented image *I*, the entropy of region *j* is defined as in Equation 9:


(9) $$H_{r}\;\left(R_{j}\right)=-\sum_{m\in V_{j}}\frac{L_{j}\,\left(m\right)}{S_{j}}\,l\,o\,g\,\frac{L_{j}\,\left(m\right)}{S_{j}}$$

where *V*_*j*_ represents the set of luminance values of all the pixels in area *j*, *L*_*j*_(*m*) represents the number of pixels with luminance value *m* in area *j*, and the region entropy of the segmented image *I* can be calculated as in Equation 10.


(10) $$H_{r}\;\left(I\right)=\sum_{j=1}\left(\frac{S_{j}}{S_{I}}\right)\,H_{r}\;\left(R_{j}\right)$$

The layout entropy of the segmented image *I* is defined as:


(11) $$H_{l}\;\left(I\right)=-\sum_{j=1}^{N}\frac{S_{j}}{S_{I}}\,\log\frac{S_{j}}{S_{I}}$$

The evaluation metric *E* can be obtained from Equations 10 and 11,


(12) $$E=H_{r}\;\left(I\right)+H_{l}\;\left(I\right)$$

The smaller the *E* value, the better the performance of the segmentation algorithm, and vice versa.

### Results and analysis

For the compared methods, the segmentation results of the k-means algorithm (Dasgupta, 2008) and FCM algorithm (Yan et al., 2012) are affected by the initial clustering centers and the initial membership matrix, respectively. Therefore, to ensure that the segmentation results remain stable when segmenting an image, for the k-means algorithm, the optimal *K* value for each image to be segmented is determined using the elbow method combined with the primary color category of the image rather than personal color preference (Huang et al., 2021). Then, the image is segmented ten times according to the *K* value. The clustering centers of each segmentation result are accumulated, and the mean value of the ten segmentation clustering centers is obtained and used as the initial clustering centers of the k-means algorithm to ensure that the segmentation results are consistent in each run.

**FIGURE 4:**
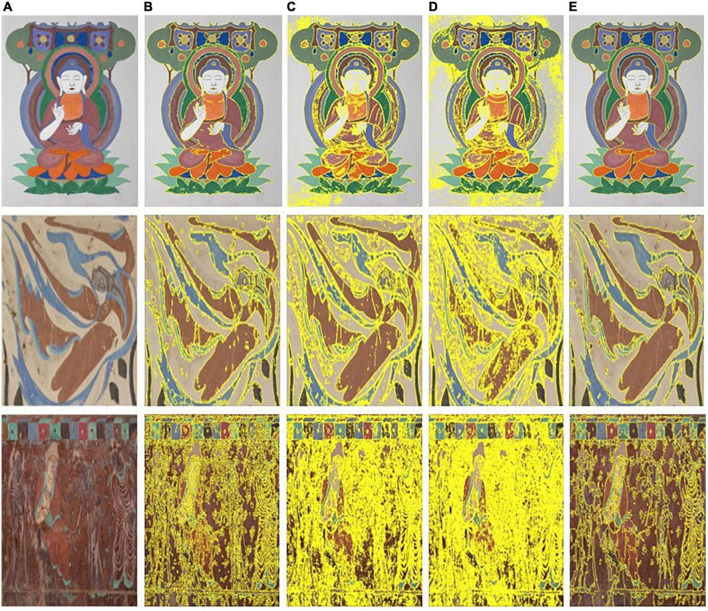
Test images and segmenting results of the different segmenting methods. **(A)** Test image. **(B)** Kovesi. **(C)** Dasgupta. **(D)** Yan. **(E)** Proposed.

For the FCM algorithm, the classification number *K* of the image to be segmented is the same as that of the k-means algorithm. Similarly, the image is segmented ten times, and the membership matrix of each segmentation result is accumulated to obtain the average value of the membership matrix of the ten repetitions, which is considered as the initial membership matrix of the algorithm. The three test images used in this study and the segmentation results obtained using the different methods are shown in Figure 4.

**FIGURE 5:**
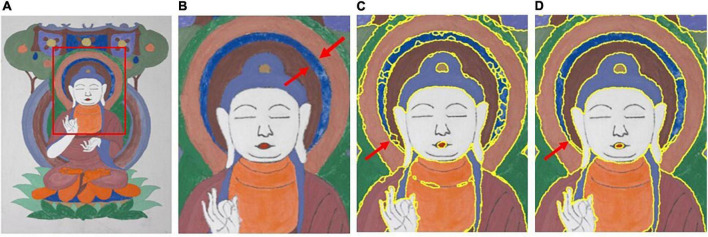
Comparison of the segmenting results between the proposed method and the method proposed by Kovesi (2013). **(A)** Test image. **(B)** ROI of test image. **(C)** Kovesi. **(D)** Proposed.

In Figure 4, from left to right, the first column shows the test images and the second to fifth columns show the segmentation results for each of the test images using the methods in the literature (Dasgupta, 2008; Yan et al., 2012; Kovesi, 2013) and the proposed method. The local segmentation results of the proposed method were compared with those reported in the literature (Kovesi, 2013). Using the first image as an example, the segmentation results are shown in Figure 5.

**FIGURE 6:**
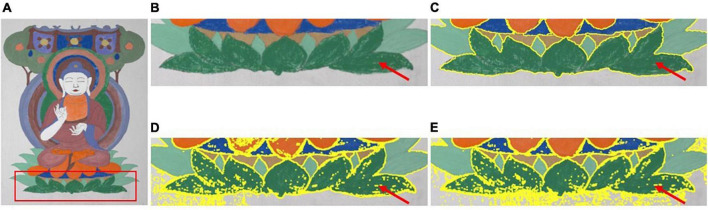
Comparison of the segmenting results between the proposed method and the methods proposed by Dasgupta (2008) and Yan et al. (2012). **(A)** Test image. **(B)** ROI of test image. **(C)** Proposed. **(D)** Dasgupta. **(E)** Yan.

It can be observed from the detailed comparison of the segmentation results indicated by the arrow in Figure 5 that the proposed method not only has a better clustering result for the superpixels, but also can process mixed-color superpixels compared with the method in the literature (Kovesi, 2013). The contrast area is well segmented by the proposed optimized method, and the color-mixing superpixels are segmented and merged into the adjacent cluster. The processed result of the mixed-color superpixels is indicated by the red arrows in Figures 5C,D. Therefore, the proposed method can eliminate the shortcomings existing in the literature (Kovesi, 2013), that is, it only performs a single clustering task and cannot process the mixed-color superpixels. In addition, the proposed method is compared with the methods in the literature (Dasgupta, 2008; Yan et al., 2012), and the results are shown in Figure 6.

From left to right, Figure 6A shows the test image and Figure 6B shows the subimage marked by the red rectangle in Figure 6A. Figures 6C–E shows the segmentation results of the compared area, as shown in Figure 6B, using the method proposed in the literature (Dasgupta, 2008; Yan et al., 2012). It can be observed from Figure 6 that the segmented results obtained using the methods proposed by Dasgupta (2008) and Yan et al. (2012) are apparently inferior to the proposed method. For the methods in the literature, the segmentation of the input image at the pixel level inevitably leads to several isolated segmented areas in the segmented image. Thus, the positional connectivity cannot be achieved for high-precision segmentation purposes. Therefore, these methods are not suitable for the fine segmentation of ancient mural images for digital restoration applications.

Fortunately, the proposed optimized segmentation method can effectively overcome the shortcomings of the existing methods in the literature (Dasgupta, 2008; Yan et al., 2012), where the mural images that have faded or worn problems are well segmented to meet the positional connectivity required in segmenting ancient mural images. The proposed method can achieve not only color consistency but also positional connectivity for a specific color and can avoid over-segmentation problems existing in the current methods. The objective evaluation metrics of the segmented results for the three tested images using the different methods are summarized in Tables 3, 4, where the best results are marked in bold. It can be observed that each row of data in Table 3 shows the evaluation metric data *F* for the same test image segmented by different segmentation methods. In the case of the same exponential level, for each test image, the value of the evaluation metric corresponding to the proposed method is obviously better than other methods, and it is the smallest. In Table 4, each row of data shows the evaluation metric *E* for the same test image segmented by different segmentation methods. Compared with other methods for each test image, the difference in metric data corresponding to all methods is very small, but the data corresponding to the method in this paper are still the smallest.

**TABLE 3:** The evaluation metric F of the tested images under different segmentation methods.

	Method			
	Kovesi	Dasgupta	Yan	Proposed
Image_1	2.2137e+07	14.577e+07	16.241e+07	**0.82274e+07**
Image_2	3.6151e+07	3.6067e+07	5.0533e+07	**1.2641e+07**
Image_3	88.342e+07	114.59e+07	127.32e+07	**26.250e+07**

**TABLE 4:** The evaluation metric E of the tested images under different segmentation methods.

	Method			
	Kovesi	Dasgupta	Yan	Proposed
Image_1	7.6955	7.9774	8.3741	**7.6043**
Image_2	8.6352	8.6821	9.1769	**8.2291**
Image_3	11.9010	11.3672	11.9276	**10.9953**

From the results of the evaluation metrics on mural image segmentation in Tables 3, 4, it can be observed that for three test images with different color complexities, the proposed optimized method always has the smallest values for the two metrics compared with the methods proposed by Dasgupta, 2008; Yan et al., 2012; Kovesi, 2013. Especially for the evaluation metric *F*, the proposed method is significantly superior to the other three methods in terms of segmenting faded or colored worn ancient mural images. Furthermore, by reviewing the visual effect of the segmented results from Figures 4-6, it can be concluded that the proposed method could have addressed the challenges in segmenting the ancient mural images to a certain extent. However, more practical applications should be conducted to ensure the effectiveness and superiority of the proposed method.

## Conclusion

Computer-aided digital restoration of the color of ancient murals is an important scientific technology, and the high-precision segmentation of ancient mural images is the foundation of digital virtual restoration. However, the complexity of the appearance of ancient murals poses a challenge for high-precision segmentation when using traditional segmentation algorithms. Therefore, an optimized image segmentation method was proposed to improve the segmentation accuracy of ancient mural images. Four key optimized strategies were developed following the SLIC and DBSCAN algorithms. They are as follows: (1) merging the small noise superpixels, (2) segmenting and merging the large noise superpixels, (3) merging the initial clusters based on their color similarity and positional adjacency to obtain the merged regions, and (4) segmenting and merging the color-mixingspecific color. It could further noisy superpixels in each of the merged regions. The proposed method could achieve not only color consistency but also positional connectivity for a specific color. It could further avoid over-segmentation problems existing in the current methods. By applying the proposed strategies sequentially to the initial clustered images, the fine-segmented mural images were obtained. The experiments verified the effectiveness and superiority of the proposed method. Additional tests will be conducted in future to further investigate and optimize the proposed method.

## Data availability statement

The original contributions presented in this study are included in the article/supplementary material, further inquiries can be directed to the corresponding author.

## Author contributions

JL was involved in methodology, data collection and analysis, and writing—reviewing. AL was involved in methodology, data collection and analysis, and writing. JZ was involved in data collection and analysis. LX and ZZ were involved in data collection. ZL was involved in data analysis and writing—reviewing. HL was involved in data analysis. JC was involved in methodology and data collection. XH was involved in methodology, funding acquisition, and writing—reviewing. All authors contributed to the article and approved the submitted version.
